# Exploring pequi (*Caryocar brasiliense* Camb.) mesocarp flour of Brazilian Cerrado biome to produce gluten‐free antioxidant biscuits

**DOI:** 10.1111/1750-3841.70070

**Published:** 2025-03-07

**Authors:** Viviane Patrícia Romani, Daniel Emanuel Cabral de Oliveira, Juliana Aparecida Célia, Adrielle Borges de Almeida, Marcos Antônio Almeida Rocha, Danihanne Borges e Silva, Osvaldo Resende

**Affiliations:** ^1^ Federal Institute of Paraná – Palmas Campus Palmas Paraná Brazil; ^2^ Goiano Federal Institute – Iporá Campus Iporá Goiás Brazil; ^3^ Goiano Federal Institute – Rio Verde Campus Rio Verde Goiás Brazil

**Keywords:** antioxidant food, functional food, gluten‐free biscuits, pequi fruit

## Abstract

**Abstract:**

The peels of pequi (*Caryocar brasiliense* Camb.), native to the Brazilian Cerrado, represent ∼80% of the mass of the whole fruit. Despite their high quantities of dietary fiber and bioactive compounds, they are generally discarded as waste. The impact of substituting rice flour with pequi mesocarp flour (PMF) in different concentrations in biscuit characteristics was determined. Pequi mesocarp was converted into flour through drying and milling, and used in quantities of 10%, 20%, and 30% in biscuit formulations. Biscuits were evaluated by their composition, physical parameters, microstructure, and antioxidant capacity. PMF was responsible for increasing protein and ash contents in biscuits. Besides that, higher concentrations of PMF resulted in a darker color of biscuits, higher spread ratio and firmness, and lower weight loss and specific volume. Such effects were attributed to the water and oil‐holding capacity because of dietary fibers present in pequi mesocarp. All biscuits produced with PMF showed antioxidant capacity, it increased with higher substitutions. Even though using PMF resulted in darker and firmer biscuits, the results indicate that PMF has the potential to produce functional gluten‐free bakery products.

**Practical Application:**

Pequi peels have a rich composition representing an opportunity to improve the nutritional attributes of bakery products. The pequi mesocarp biscuits produced are gluten‐free and contain antioxidants, promoting consumers' health. Producing biscuits with pequi mesocarp is advantageous to offer the population a nutritive alternative biscuit, while adding value to pequi peels, strengthening the local economy, and decreasing environmental pollution.

## 1 INTRODUCTION

Large amounts of food are wasted in the world every day and despite the increase of food waste valorization in the last years, few food byproducts are being converted into value‐added products. Some examples of industrially processed byproducts include cheese whey, apple pomace, citrus peels, and olive mill waste (Leão et al., 2017). Such agro‐industrial residues have been used for different purposes; for example, in the substitution of wheat flour in bakery products and to generate innovative food formulations. In the literature, there are reports regarding food byproducts/waste valorization. Wang et al. (2023) developed a protein‐enriched biscuit based on oat‐milk byproduct fortified with chickpea flour. Nogueira Soares Souza et al. (2022) utilized cocoa bean shell powder as a substitute for wheat flour on chocolate cake. Bölek (2021) studied the effects of waste fig seed powder on quality in biscuit formulation. Usage of food byproducts or waste is advantageous due to the enhancement of the nutritional quality of bakery and other food products. The main reason is that most agro‐industrial sources are rich in fibers, proteins, and bioactive compounds. Also, because of the integral use of raw materials provided by nature and the reduction of their disposal (Blicharz‐Kania et al., 2023; Dutta et al., 2023; Egea et al., 2023). Regarding fruits and vegetables, Ayala‐Zavala et al. (2011) highlighted that the quantity of bioactive substances present in byproducts/wastes may be superior to the edible part of the fruit. Fruit wastes were proposed as a source of various components, as in the case of açaí seeds (Romani et al., 2022), watermelon peels (Guo et al., 2021a, 2021b), tomato peels (Ninčević Grassino et al., 2020), grape seeds (Tami et al., 2022), among others.

Pequi (*Caryocar brasiliense* Camb.) fruit is native to the Brazilian Cerrado, and contributes significantly to the economy of producing regions. It has high quantities of dietary fiber and various bioactive compounds, known to promote consumer health. According to Leão et al. (2017), the fiber content of the pequi mesocarp is 43.3%. The edible part is the pulp, a thin layer around the almond, representing only ∼10% of the fruit. The peels (exocarp and mesocarp), which correspond to ∼80% of the mass of the whole fruit, are generally discarded as waste (Gonçalves Martins et al., 2021; Moreira et al., 2019). Ramos and Souza (2011) evaluated the characteristics of 180 pequi fruits obtained from 36 plants and reported that the mass of the mesocarp represents around 77.28%, the average mass of the almond is 14.94%, the average mass of the pulp is 6.72%, and the mass of the almond is 1.06%. Similar results were reported by Gomes et al. (2022) when characterizing 170 pequi fruits.

Pequi peels have been studied and considered to produce value‐added products due to the large amounts of pectic polysaccharides (Moreira et al., 2019). Brito Cangussu et al. (2021) characterized the pequi flour and detected bioactive compounds as flavonoids, gallic acid, ellagic acid, lutein, β‐carotene, gallate ethyl, β‐cryptoxanthin, tannins, and saponins. They confirmed the multifunctional potential of pequi peel flour as a functional ingredient. Other studies further reported that pequi mesocarp flour (PMF) has antioxidant and antimicrobial substances such as tannins, lectin, coumarins, triterpenes, steroids, and essential oil (do Nascimento et al., 2017; Leão et al., 2017; Moreira et al., 2019). Leão et al. (2017) revealed that pequi peels have ∼56% pectic polysaccharides with application as food ingredients (e.g., thickener, gelling agent, and emulsifier). These components make pequi peels attractive for producing nutritional and value‐added products while generating value for an agro‐industrial residue and preventing environmental pollution.

Gluten‐free bakery products are in high demand for various reasons, including celiac disease, gluten sensitivity, and the interest in anti‐inflammatory diets. Among them, chemically leavened bakeries such as crackers, biscuits, and cakes are receiving increasing attention because they offer convenience and exclusive sensory attributes (Pan et al., 2020; Schouten et al., 2024; Yegin et al., 2024). Substitutes of wheat flour to produce these products include rice, corn, tapioca, potato, soy, amaranth, quinoa, and other flours (Silva & Conti‐Silva, 2016; Zhao et al., 2023).

The drying operation of pequi peels has been studied to generate flour for food products (Souza et al., 2019), by the flour properties and its composition (Costa et al., 2017; Leão et al., 2017; Soares Júnior et al., 2010), and some applications in foods (e.g., jelly; Siqueira et al., 2012), wheat biscuits; (Soares‐Junior et al., 2009), packaging (Guerra et al., 2023; Siqueira et al., 2022), and other sectors (e.g., energy generation; Ghesti et al., 2022; Gonçalves Martins et al., 2021). Leão et al. (2017) reported that flours prepared from pequi byproducts are a promising food ingredient, with high levels of dietary fiber, phenolics, and carotenoids. However, despite the nutritional and technological potential of pequi peels, their use to develop new products or as a source of compounds is still limited. To the best of our knowledge, pequi mesocarp was not used to produce gluten‐free bakery products. Thus, the present study aimed to identify the impact of substituting different concentrations of rice flour with PMF on biscuit composition and characteristics.

## 2 MATERIAL AND METHODS

### 2.1 Material

Pequi fruits (*Caryocar brasiliense* Camb.) from Iporá, GO, Brazil, harvested in 2022 were obtained in the local market. The fruits were collected, selected, and stored under refrigeration (∼7°C) in the Laboratory of Postharvest and Processing at Goiano Federal Institute (IF Goiano, Iporá Campus). All the ingredients for biscuit preparation were purchased in the local market: rice flour (Urbano), sugar (Cristal Alimentos), butter (Piracanjuba), eggs (Granja Primavera), and baking powder (Royal). 2,20‐Azino‐bis(3‐ethylbenzothiazoline‐6‐sulfonic acid) diammonium salt (ABTS) and 1,1‐diphenyl‐2‐picryl‐hydrazyl (DPPH) were purchased from Sigma‐Aldrich. Potassium persulfate was purchased from Êxodo Científica. The chemicals used for analysis were all of analytical grades.

### 2.2 Production and technological characterization of pequi mesocarp flour

Pequi fruits were selected based on the absence of visual injuries or infections. They were cleaned with neutral detergent, washed with tap water, and dried with a paper towel. To produce the mesocarp flour the fruits were cut and the mesocarp was separated from the epicarp and endocarp. The mesocarp was cut into thin slices (2–4 mm) using a stainless‐steel knife and bleached in boiling water for 3 min. The drying process was performed as described by Siqueira et al. (2022) in an oven with forced air circulation (Ethik Technology, 400‐4D) at 60°C/12 h. Dried pequi mesocarp was grounded using a knife mill (Solab, SL‐32) to generate the flour. The granulometry of the flour was standardized using a sieve (35 mesh, 500 µm). The flour was stored in dark containers under refrigerated conditions until use.

PMF was analyzed by its oil‐holding capacity (OHC) and water‐holding capacity (WHC) and compared to rice flour, following the methodology of Shad et al. (2013). For WHC 1 g of flour was vortexed with 10 mL of distilled water in preweighed centrifuge tubes for 30 min. This mixture stood at room temperature for 30 min before centrifugation (Novatecnica, NT 812) at 3000 × *g* for 25 min. Then, the supernatant (water) was removed, and the sediments were weighed (BEL, M214AI). For OHC, 0.5 g of the flour samples were weighed, mixed with 5 mL of canola oil, and proceeded further as done for WHC. The WHC (Equation 1) and OHC (%; Equation 2) were calculated as described in Equation (1), where *W*_0_ corresponds to the flour weight, *W*_1_ is the centrifuge tube weight + flour, and *W*_2_ is the centrifuge tube weight + sediments.

(1) $$\mathrm{WHC}\left(\%\right)=\left[\left(W_{2}-W_{1}\right)/W_{0}\right]$$

(2) $$\mathrm{OHC}\left(\%\right)=\left[\left(W_{2}-W_{1}\right)/W_{0}\right]$$

### 2.3 Biscuits preparation

Gluten‐free rice biscuits were produced following the method described by Silva and Conti‐Silva (2016) with adaptations. Control biscuits were prepared using rice flour, sugar, butter, eggs, and baking powder. The rice flour was then replaced by PMF (10%, 20%, and 30%) according to Table 1. The concentrations of PMF used were determined based on preliminary tests. To produce biscuits, sugar, butter, and eggs were mixed in a Philco mixer (Planetary PHP 500 Turbo) at speed 2 for 3 min. Then flour and baking powder were mixed and added to the sugar, butter, and eggs. This mixture was then mixed at speed 1 for 3 min. The dough was rolled to 1 cm, cut into biscuits having 5.5 cm diameter using a mold, and baked at 180°C for 15 min in an oven (Fisher, Hot grill). The biscuits were cooled to room temperature, packed in sealed plastic bags, and kept at room temperature overnight before analysis.

**TABLE 1:** Ingredients utilized to prepare rice and pequi mesocarp biscuits and their quantities.

Ingredient	Quantity			
	C	F1	F2	F3
Rice flour	190 g	171 g	152 g	114 g
Pequi mesocarp flour	0 g	19 g	38 g	76 g
Sugar	90 g	90 g	90 g	90 g
Butter	50 g	50 g	50 g	50 g
Eggs	50 g	50 g	50 g	50 g
Baking powder	5 g	5 g	5 g	5 g

C, control rice biscuits; F1, biscuits with 10% of pequi mesocarp flour; F2, biscuits with 20% of pequi mesocarp flour; and F3, biscuits with 30% of pequi mesocarp flour.

### 2.4 Proximal composition of flours and biscuits

Water activity (*a*_W_) was measured with a HygroPalm (AW1, Rotronic, Bassersdorf) at 25 ± 1°C. Proximal composition of biscuits produced, PMF and rice flour was determined according to the methods of the Association of Official Analytical Chemists International (AOAC, 2023). Moisture content was based on water evaporation in an oven at 105°C, protein content by determining total nitrogen using the Kjeldahl method, lipid content using the extraction method by Soxhlet with petroleum ether, and ash by incineration in a muffle furnace at 550°C. The total carbohydrate content was determined by difference, subtracting the sum of moisture, protein, lipid, and ash percentages from 100.

### 2.5 Physical and textural properties of biscuits

The diameter (mm) and thickness (mm) of biscuits were determined with a caliper (Zaas Precision, Amatools^®^) at three different points and expressed as means. The spread ratio was calculated by dividing diameter by thickness (Akesowan, 2016). Weight loss (%) of the biscuit was determined based on the initial weight (mo), weight after baking (mt) and calculated as mo − mt divided by mo (Lara et al., 2011).

The specific volume (cm^3^ g^−1^) was measured by rapeseed displacement method according to AACC (2010). The specific volume was calculated by dividing the volume by the weight of each biscuit analyzed. The expansion factor was also determined according to AACC (2010), calculated as the diameter/thickness of the biscuit after baking divided by the diameter/thickness of the biscuit before baking.

The hardness of biscuits was determined with a CT3 Texture Analyzer (Brookfield Engineering Labs. Inc.) fitted with a TA 4/100 flat bottom probe (38.46 mm in diameter). Nine biscuits were analyzed for each formulation. It used a 25 kg load cell, a distance of 191.5 mm, and pretest, test, and post‐test speeds of 2 mm s^−1^, 2 mm s^−1^, and 4.5 mm s^−1^, respectively. The peak force (*N*) needed to break the biscuit was recorded by the CT3 texture analyzer coupled to the Texture Pro CT V1.4 Build 17 program (Brookfield Engineering Labs. Inc.). Color measurement of PMF and biscuits was performed using a Hunter colorimeter fitted with an optical sensor (ColorFlex EZ, Hunter Associates Laboratory Inc.) in terms of CIE L* (brightness/darkness), a* (redness/greenness), b* (yellowness/blueness) color system. The chroma value (C*) and hue were determined using a* and b* values (Schouten et al., 2024).

### 2.6 Antioxidant capacity

The procedure for extracting antioxidants and evaluating antioxidant capacity was according to Rufino et al. (2010). Approximately 1 g of each sample was weighed and 40 mL of 50% (v/v) methanol was added. This mixture was homogenized and rested for 1 h at room temperature. Then, it was filtered and transferred to a volumetric flask. From the residue from the first extraction, 40 mL of 70% (v/v) acetone was added, homogenized, and remained for 1 h at room temperature. It was filtered, the supernatant was transferred to the volumetric flask containing the first filtrate and topped up the volume to 100 mL with distilled water for analysis.

The antioxidant capacity was determined by the DPPH and ABTS^+^ methods, both to quantify free radical scavenging. For the DPPH method, a methanol solution containing 0.06 mM DPPH was prepared. The blank was adjusted with methanol, and then an aliquot of 100 µL of sample extract was added to 3.9 mL of this solution. The decrease in absorbance at 515 nm (UV‐5100, Metash) was measured after 30 min. Results were expressed as the percentage of the radical oxidation inhibition with the control. For the ABTS^+^ assay, ABTS^+^ radical cations were initially produced using 7 mM of ABTS stock solution mixed with 145 mM potassium persulfate. This mixture remained in the dark at room temperature for 12–16 h. Before its use, the ABTS^+^ solution was diluted with ethanol to an absorbance of 0.70 ± 0.02 at 734 nm. After adding 30 µL of sample or Trolox standard to 3 mL of diluted ABTS^+^ solution, absorbances were recorded at 6 min after mixing. Ethanolic solutions of known Trolox concentrations were used for calibration and the results were expressed as µM Trolox g^−1^ sample.

### 2.7 Scanning electron microscopy

Scanning electron microscopy (SEM) images of the different biscuits’ formulations were obtained on a Jeol JSM7100F field emission scanning electron microscope (SEM‐FEG). An electron accelerating voltage of 5 keV was used in secondary electron detection (SED) modes. Biscuit samples were placed into aluminum stubs using double‐sided tape, plated with a thin gold film (10 nm), and examined at magnifications 1000× and 3000×.

### 2.8 Statistical analysis

Completely randomized design and one‐way factorial analysis of variance (ANOVA) were performed using Statistica 7.0 software followed by the Tukey's test at a significance level of 5% (*p* < 0.05). All analyses were performed in triplicate.

## 3 RESULTS AND DISCUSSION

### 3.1 Pequi mesocarp flour

The flour composition in a bakery is of utmost importance because it is directly related to the product characteristics. The proximal composition of PMF was determined and presented in Table 2. Protein and ash contents of PMF were similar to those reported by Soares Júnior et al. (2010), which obtained 5.59% and 2.86%, respectively. Lipids and moisture were higher in the flour of the present study in comparison to the values reported (0.85% and 3.08%, respectively; Soares Júnior et al., 2010). Differences among the values reported in the literature and the present study were expected because fruits have variable composition depending on different factors (e.g., species, cultivars, place of production, soil and climate, cultural practices, degree of maturity, and harvest time).

**TABLE 2:** Proximal composition (g/100 g) of pequi mesocarp flour and rice and pequi mesocarp flour biscuits.

Analysis	Sample				
	PMF	C	F1	F2	F3
*a*_W_	0.474 ± 0.003	0.617 ± 0.003^b^	0.641 ± 0.004^a^	0.502 ± 0.002^d^	0.535 ± 0.006^c^
Moisture	13.82 ± 0.18	9.12 ± 0.09^b^	9.71 ± 0.06^a^	8.07 ± 0.17^c^	9.17 ± 0.17^b^
Proteins	5.33 ± 0.45	5.81 ± 0.29^b^	6.62 ± 0.23^a^	6.57 ± 0.06^a^	6.26 ± 0.03^ab^
Lipids	1.61 ± 0.07	14.73 ± 0.31^a^	14.92 ± 0.59^a^	16.78 ± 1.40^a^	16.14 ± 2.63^a^
Ash	2.46 ± 0.45	0.85 ± 0.02^d^	0.95 ± 0.01^c^	1.08 ± 0.02^b^	1.19 ± 0.02^a^
Carbohydrates*	76.78 ± 0.35	69.49 ± 0.21^a^	67.80 ± 0.94^a^	67.50 ± 1.59^a^	67.24 ± 3.14^a^

PMF, pequi mesocarp flour; C, control rice biscuits; F1, biscuits with 10% of pequi mesocarp flour; F2, biscuits with 20% of pequi mesocarp flour; and F3, biscuits with 30% of pequi mesocarp flour. Different letters in the same line indicate significant differences among biscuit samples (*p* < 0.05) by the Tukey's test. *Resulting in 100 – (moisture + protein + lipid + ash).

The composition of PMF is close to rice flour in terms of proteins (8.78%), lipids (2.62%), and ash (0.65%), with the added advantage of having active compounds (e.g., antioxidants). Antioxidant activity assays were performed, and the active potential of PMF was confirmed. DPPH assay showed 94.3 ± 0.4% of radical scavenging capacity, while ABTS presented 2281.44 ± 4.16 µM Trolox g^−1^ sample. This composition confirms the potential of PMF to generate functional foods, such as bakery products and especially gluten‐free options.

Water activity is an important parameter in controlling microorganisms and the degradation of foods. For pathogenic bacteria growth, a minimum of 0.83 is generally required (Jin et al., 2019). PMF presented *a*_W_ of 0.474 (Table 2), characterizing it as a shelf‐stable product. Around this range of water activity, most degradation reactions (e.g., lipid oxidation and enzyme activity) are reduced (Schmidt, 2004).

**FIGURE 1:**
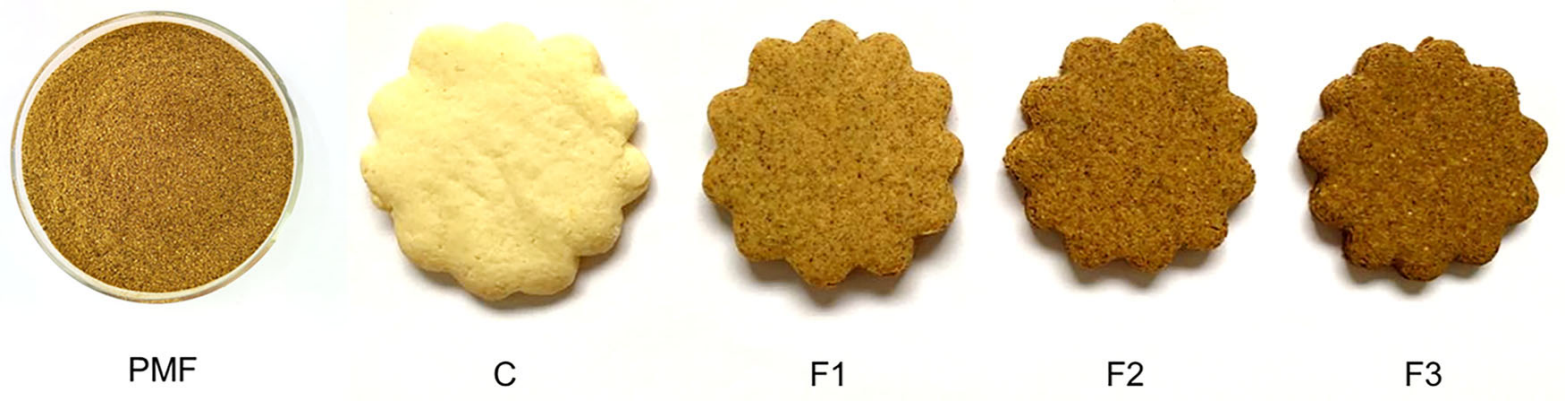
Visual aspect of pequi mesocarp flour (PMF) and biscuits made using rice flour (C) and containing PMF in the concentrations of 10% (F1), 20% (F2), and 30% (F3).

Color parameters for PMF were *L* = 48.39 ± 0.06, a* = 8.25 ± 0.14 and b* = 26.26 ± 0.18. They represent medium luminosity, and tons of yellow (+b*) and red (+a*). The hue angle was 72.56° and the chroma was 27.52. These values are similar to those reported by Leão et al. (2017), which obtained *L* of 45.2 and 55.2, the hue of 70.2° and 72.6°, and chroma of 28.9 and 30.6 for flours produced using pequi mesocarp + exocarp and only mesocarp, respectively. The produced flour visually presented a light brown coloration, as demonstrated in Figure 1. This color is characteristic of a yellowish hue (around 70°).

WHC and OHC are crucial in bakery formulations, affecting hydration and interaction with other ingredients; they are related to the texture of a product and other physical characteristics (Shen et al., 2022; Sudha, 2011). WHC and OHC of PMF were 438.29% and 138.59%, respectively, while rice flour presented lower values, 160.27% for WHC and 104.18% for OHC. As mentioned by Wang et al. (2023), high WHC is suitable for biscuits, suggesting that the PMF of the present study is adequate for this purpose. Similar values were reported by Costa et al. (2017), who obtained WHC of 450.00% and OHC of 173.33% for PMF. The lower values obtained for WHC and OHC of rice were also within the range reported in previous studies (Kakar et al., 2022).

### 3.2 Biscuits composition

Control biscuits and biscuits incorporated with different quantities of PMF were characterized by their composition. Results of water activity, moisture, proteins, lipids, ash, and carbohydrates are presented in Table 2. Most of the components were affected by the incorporation of PMF. Water activity was lower especially in higher concentrations of mesocarp flour (20% and 30%) because free water (represented by *a*_W_) is low for this flour (0.474) and due to its higher WHC compared to rice flour. Even moisture presented variations among the samples, such changes were not linear with the increase in concentrations of PMF. Proteins were significantly higher (*p* < 0.05) for the biscuits containing PMF than rice flour biscuits. Lipids were similar for all treatments; this was expected due to the amount of butter used in the composition of biscuits and the low quantity of lipids in PMF (1.61%). Ash concentrations increased as the quantity of PMF increased. This occurred due to the ash content of PMF (2.46%). Protein and ash contents of biscuits are comparable to the study of Wang et al. (2023), which produced protein‐enriched biscuits based on oat‐milk byproduct fortified with chickpea flour. Superior protein and ash contents in higher PMF formulations are advantageous mainly in nutritional aspects. Carbohydrate contents were significantly similar (*p* > 0.05) for all samples. It is important to highlight that fruit peels are known for having significant fiber content. Pequi mesocarp, for example, is rich in pectin, which belongs to dietary fibers. They are beneficial for their nutritive and health‐protective effects (Ayala‐Zavala et al., 2011). In addition, dietary fibers have an important role in the physical characteristics of biscuits due to their WHC. Thus, it was suggested by the biscuit's composition that PMF can be used to produce traditional and gluten‐free biscuits.

### 3.3 Effects of pequi mesocarp flour on physical characteristics of biscuits

Appearance of bakery products is one of the quality indicators perceived by consumers that is essential for acceptability (Wang et al., 2021). The appearance of control rice biscuits and biscuits incorporated with pequi mesocarp are shown in Figure 1. It shows that the color and volume of the biscuits were affected by the concentration of PMF added. It is worth mentioning that biscuits containing PMF were easier to shape due to the consistency of the dough. It might be related to the capacity of dietary fibers and proteins to retain water (Echeverria et al., 2022), confirmed by the WHC of PMF. In addition, as the PMF content increased in formulations, biscuits became visually darker. This characteristic was generated by the tons of yellow (+b*) and red (+a*) discussed in the color analysis of PMF, and it can be related to its higher ash content. Biscuits containing PMF appeared similar to whole grain biscuits due to their brownish color, which might be seen as positive by consumers who seek healthier foods. However, consumer acceptance needs to be further studied to confirm it.

**FIGURE 2:**
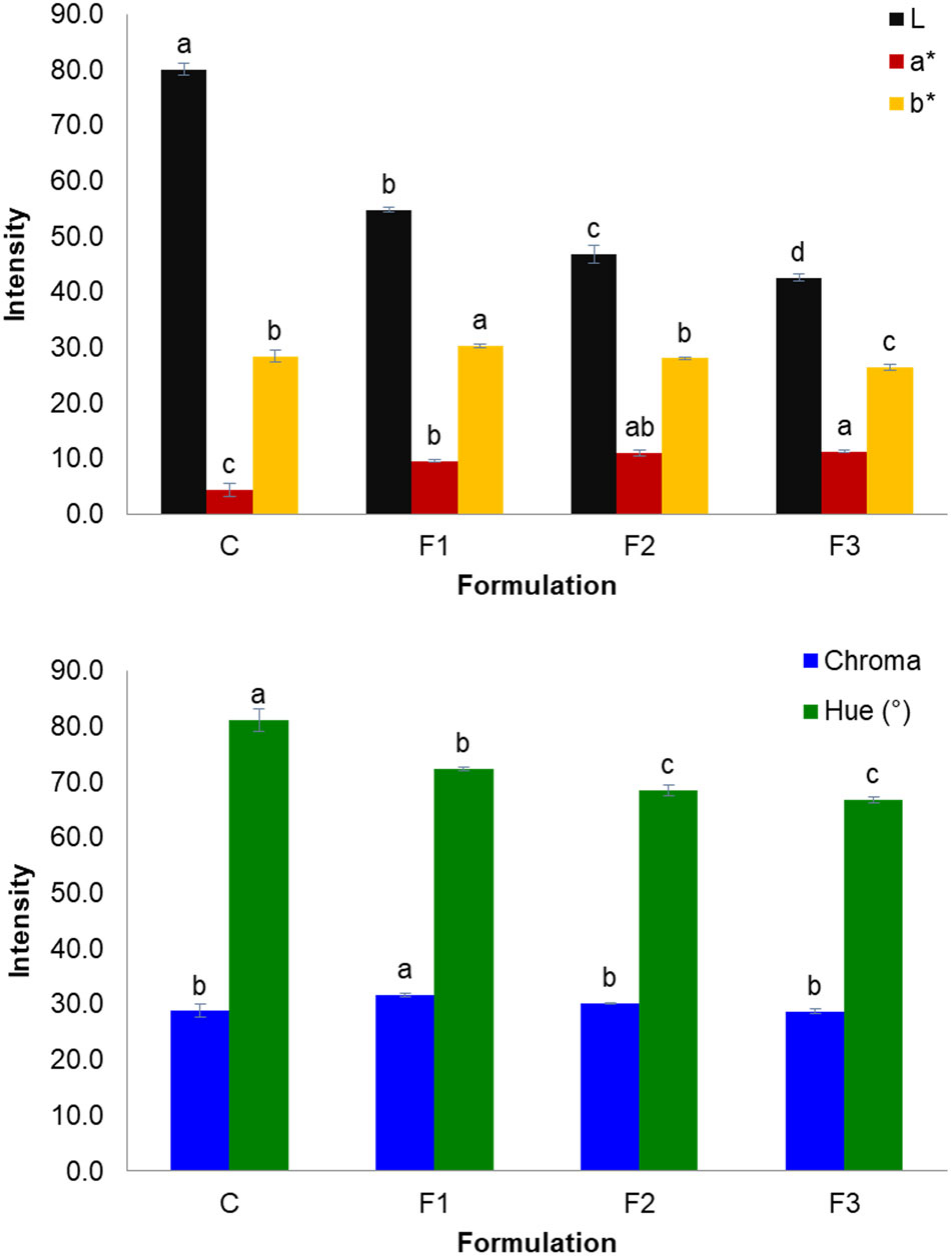
Color parameters of rice and pequi mesocarp flour biscuits. C, control rice biscuits; F1, biscuits with 10% of pequi mesocarp flour; F2, biscuits with 20% of pequi mesocarp flour; and F3, biscuits with 30% of pequi mesocarp flour. Different letters indicate significant differences among formulations (*p* < 0.05) by the Tukey's test.

The differences observed in Figure 1 were confirmed by color parameters (Figure 2) of rice and PMF biscuits. The incorporation of PMF significantly reduced (*p* < 0.05) the luminosity (*L*) of biscuits since they became darker, in agreement with visual observations. Ashes increased as PMF concentration was higher (Table 2), which might have contributed to the darker color of the biscuits. Fibers also contributed to the darker color. Duta and Culetu (2015) reported that oat bran cookies presented similar behavior; a high amount of dietary fiber generated darker and more brittle cookies. Luminosity values are comparable to whole grain biscuits (around 50), as Filipčev et al. (2012) obtained for ginger nut biscuits with wholegrain buckwheat and rye flour. Chromaticity for all the treatments in the present study had positive values for a* (red) and b* (yellow). Significant changes (*p* < 0.05) in chromaticity were observed for the different concentrations of PMF in biscuits and compared to control samples. This is highlighted by the increase in yellow tons (due to the superior b* values) for biscuits produced with 10% of PMF and red tons (due to the superior a* values) for F2 and F3. Besides the flour color effects, the darker coloration of the biscuits might have resulted from the Maillard reaction. This is characterized by the reaction of proteins with reducing sugars to form brown pigments (melanoidins; Alais & Linden, 1991). In line with the trend observed for luminosity, a* and b* values of F1, F2, and F3 were close to the obtained by Filipčev et al. (2012) for wholegrain biscuits.

Hue values are related to the luminosity, indicating that rice biscuits containing PMF are significantly darker (lower hue; *p* < 0.05) than pure rice biscuits (higher hue). Studies of rice biscuits incorporated with fruit byproducts/wastes for comparison of color were not found. Indeed, it was already expected to have a more pronounced brown color for biscuits containing PMF because of the flour color. Even though differences were observed for a* and b* values, color saturation (represented by chroma) was similar for most treatments (*p* > 0.05). In the present study, hue values were superior (∼70–80°) than presented by Akesowan (2016) (∼60–70°) in gluten‐free cookies produced using konjac flour and milk protein concentrate.

Table 3 shows that the physical parameters of biscuits were affected by PMF utilization. It can be observed that diameter and thickness decreased as the concentration of PMF increased, similar to the trend reported by Akesowan (2016). In contrast, the spread ratio was greater for biscuits containing PMF than for control biscuits. The superior spread ratio (represented by the ratio of diameter and height) suggests a higher resistance of the dough to flow. It is related to the water absorption of the biscuit's components (Kissell & Yamazaki, 1975), in line with the WHC result for PMF. Indeed, formulations containing higher PMF (F2 and F3) had a superior spread ratio. Giuberti et al. (2017) mentioned that biscuits with a higher spread ratio are considered the most desirable. These authors obtained spread ratio values of around 5.0 for gluten‐free rice crackers.

**TABLE 3:** Physical parameters of rice and pequi mesocarp flour biscuits.

	Formulation			
Parameter	C	F1	F2	F3
Diameter (mm)	62.37 ± 2.33^a^	60.29 ± 1.40^ab^	58.43 ± 0.62^b^	58.15 ± 1.47^c^
Thickness (mm)	10.99 ± 0.54^a^	10.27 ± 0.20^a^	8.35 ± 0.67^b^	8.73 ± 0.60^b^
Spread ratio	5.70 ± 0.46^c^	5.87 ± 0.20^bc^	7.04 ± 0.60^a^	6.69 ± 0.44^ab^
Weight loss (%)	11.4 ± 0.2^a^	10.2 ± 0.4^b^	9.8 ± 0.3^b^	9.9 ± 0.5^b^
Specific volume (cm^3^ g^−1^)	1.96 ± 0.06^a^	1.69 ± 0.05^ab^	1.55 ± 0.18^b^	1.50 ± 0.22^b^
Expansion factor	0.90 ± 0.05^a^	0.90 ± 0.06^a^	0.97 ± 0.06^a^	0.91 ± 0.05^a^
Hardness (N)	24.0 ± 2.6^d^	48.2 ± 1.5^c^	83.7 ± 4.4^b^	124.5 ± 9.7^a^

C, control rice biscuits; F1, biscuits with 10% of pequi mesocarp flour; F2, biscuits with 20% of pequi mesocarp flour; and F3, biscuits with 30% of pequi mesocarp flour. Different letters in the same line indicate significant differences among samples (*p* < 0.05) by the Tukey's test.

When PMF was used, weight loss reduced significantly (*p* < 0.05). The weight loss reduction resulted from the superior water retention capacity of PMF. It occurs due to proteins and dietary fiber in its composition, making the cookies lighter and harder, as also reported by Echeverria et al. (2022). Fibers and proteins tend to retain moisture in the dough, which not only reduces the water content but can also affect the lightness of the product. They provide a more rigid structure, which can contribute to a crispier and lighter texture. Additionally, dietary fibers, such as the pectic polysaccharides, of pequi mesocarp can bind water (dos Santos & Grenha, 2015; Leão et al., 2017). Then, less free water was available as confirmed by *a*_W_, impacting the physical properties of biscuits.

Specific volume significantly reduced (*p* < 0.05) as the content of PMF increased, mainly for 20% and 30% of the substitution of rice flour by PMF (Table 3). Specific volume is a consequence of air bubbles retaining. Air bubbles are generated during baking, which increases the biscuit volume (Akesowan, 2016). When PMF was incorporated, it might have impacted the capacity of the dough to retain air bubbles, and/or the superior dough viscosity prevented dough expansion. In addition, this reduction might have occurred due to the lower free water available (confirmed by lower *a*_W_), impacting the reactions of baking soda (chemical leavener), which require water to release gas. The specific volume of the biscuits in the present study was similar to the one reported by Silva and Conti‐Silva (2018) for a wheat chocolate commercial cookie. This similarity indicates that the volume of all biscuits is suitable for commercialization. The expansion factor is another physical phenomenon controlled by water absorption (Kissell & Yamazaki, 1975). Even though the water absorption of PMF was indicated as the reason for the differences observed for other parameters, the expansion factor was not significantly affected (*p* > 0.05).

Texture (hardness and crispness) is among the critical quality indicators for consumers' acceptability. The moisture content and distribution largely influence it in the product (Wang et al., 2021). A significant increase (*p* < 0.05) in the hardness of biscuits was observed as PMF replaced the quantity of rice flour. This might be another effect of the water absorption of the flour due to the presence of dietary fibers that bind water. Other authors (Paucean et al., 2016; Xu et al., 2020) explained that such changes can result from the number of hydrophilic sites available to compete for the limited free water, suggesting that higher moisture addition is necessary. Furthermore, some authors attribute higher hardness to the interaction of proteins and polysaccharides by hydrogen bonding (Sarabhai et al., 2015). Silva and Conti‐Silva (2016) observed the same effect when wheat flour was replaced by rice flour, whole soy flour, and starch in chocolate cookies. Ktenioudaki and Gallagher (2012) explained that dietary fibers affect baked product characteristics, and modifications include higher crumb hardness, crispness loss, and changes to appearance and flavor. Giuberti et al. (2017) obtained values around 60–70 N for hardness of rice cookies, between the values of F1 and F2 formulations of the present study. Silva and Conti‐Silva (2018) reported the physical properties of a commercial cookie and presented 43.3 N for hardness, which is close to the hardness obtained for F1 in the present study. Then, among the formulations of the present study, F1 (10% of PMF) can be suggested as the most adequate according to the physical parameters, since the superior hardness of F2 and F3 could be considered a drawback compared to control rice biscuits and F1.

### 3.4 Microstructure of the different biscuits

**FIGURE 3:**
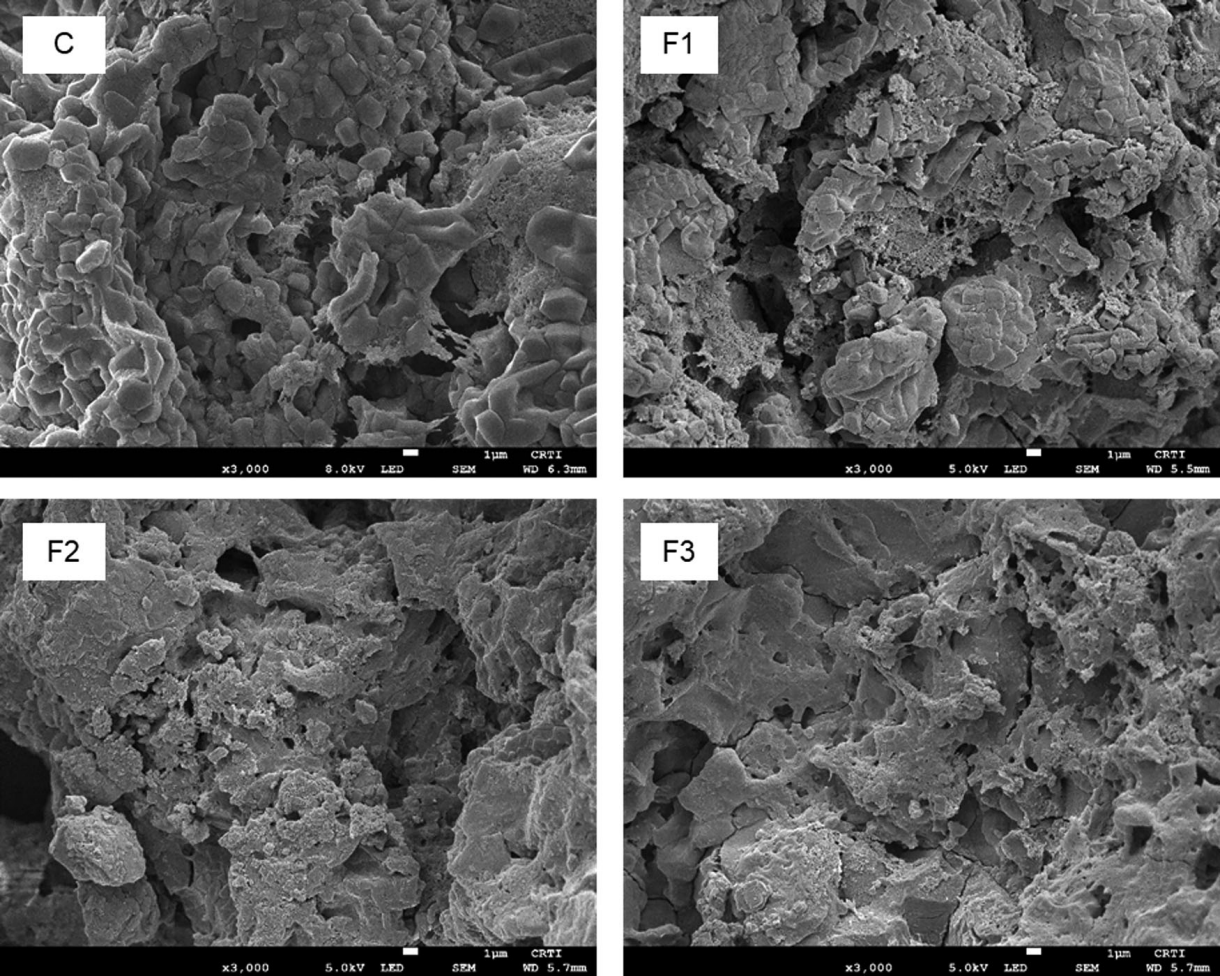
Scanning electron microscopy (SEM) of cookies produced with different formulations. C, control rice biscuits; F1, biscuits with 10% of pequi mesocarp flour; F2, biscuits with 20% of pequi mesocarp flour; and F3, biscuits with 30% of pequi mesocarp flour.

SEM was used for microstructure observations to investigate the effects of PMF addition in rice biscuits due to the influence of structural changes on physicochemical properties. Figure 3 shows the microstructure of control rice biscuits and biscuits incorporated with PMF. Control rice biscuits clearly showed the presence of intact rice starch granules with characteristic smooth surfaces, and angular and polygonal shapes, as Shimizu and Ushiyama (2020) previously described. These starch granules were connected to a network formed by fat and proteins. With the incorporation of PMF in rice starch biscuits, the amount of starch granules reduced in each formulation considering the substitution quantity (F1, F2, and F3). It was also observed that increasing the amount of PMF formed a more compact and uniform structure (F2 and F3). Different from rice flour, PMF has irregular shapes and sizes (Figure S1). Part of its particles might have melted and contributed to the formation of a network together with the other components of formulations. These observations follow the results of physical parameters, where there was a significant decrease (*p* < 0.05) in specific volume and an increase in hardness (related to WHC).

### 3.5 Impact of pequi mesocarp flour on antioxidant properties of biscuits

Biscuits produced with PMF presented antioxidant activity (Table 4) and increased according to the percentage of PMF. DPPH and ABTS^+^ assay results showed that incorporating PMF resulted in a superior antioxidant capacity (*p* < 0.05). Both methods used are radical scavenging assays; antioxidants donate a hydrogen atom to the corresponding hydrazine, reducing the odd electron of the nitrogen atom in DPPH (Kedare & Singh, 2011), while for ABTS^+^ is suggested the mixed mechanism of hydrogen atom transfer/single electron transfer, and concerted electron‐proton transfer, among other mechanisms (Ilyasov et al., 2020). Then, all the PMF‐biscuits demonstrated to work as antioxidants through different mechanisms of action. It is important to note that for F3 (containing 30% of PMF), the radical scavenging activity was close to the pure PMF (94.3%) according to the DPPH assay.

**TABLE 4:** Antioxidant activity of rice and pequi mesocarp flour biscuits.

	Antioxidant assay	
Formulation	1,1‐Diphenyl‐2‐picryl‐hydrazyl (DPPH; %)	2,20‐Azino‐bis(3‐ethylbenzothiazoline‐6‐sulfonic acid) diammonium salt (ABTS; µM Trolox g^−1^ sample)
C	3.7 ± 2.3^d^	144.78 ± 12.27^d^
F1	38.0 ± 5.3^c^	520.33 ± 22.28^c^
F2	85.7 ± 3.2^b^	1415.89 ± 15.71^b^
F3	92.7 ± 1.3^a^	1909.22 ± 49.62^a^

C, control rice biscuits; F1, biscuits with 10% of pequi mesocarp flour; F2, biscuits with 20% of pequi mesocarp flour; and F3, biscuits with 30% of pequi mesocarp flour. Different letters in the same line indicate significant differences among samples (*p* < 0.05) by the Tukey's test.

The antioxidant activity observed for biscuits containing PMF resulted from bioactive compounds present in pequi mesocarp. Among these components are the ones identified by Brito Cangussu et al. (2021), including flavonoids, gallic acid, gallate ethyl, ellagic acid, lutein, β‐carotene, and β‐cryptoxanthin. Leão et al. (2017) also reported that pequi byproducts (flour corresponding to the exocarp and the mesocarp) have important quantities of total polyphenols, nonextractable proanthocyanidins, and carotenoid associated with dietary fiber responsible by health benefits, as they are nutraceutical.

From Table 4 results, it is confirmed that the functionality of the bioactive compounds from PMF was preserved after the baking of biscuits. Then, all the formulations containing PMF are functional biscuits and might contribute to consumers' health. It is important to highlight that the reported antioxidant capacity is advantageous not only for consumption but contributes to the shelf life of biscuits due to the prevention of lipid oxidation, which is one of the main causes of food spoilage.

## 4 CONCLUSION

Pequi mesocarp was transformed into flour and presented the potential to produce bakery products. Its composition has proteins, carbohydrates, ashes, and important quantities of antioxidants and dietary fibers responsible for functional properties. Replacing rice flour with pequi mesocarp in concentrations of 10%, 20%, and 30% significantly affected the gluten‐free biscuits' characteristics, including composition, physical parameters, microstructure, and antioxidant capacity. It was observed that higher quantities of PMF resulted in higher contents of proteins and ashes, darker color, higher spread ratio and hardness, and lower weight loss and specific volume. Most of the effects were attributed to the superior WHC and OHC of PMF due to the proteins and dietary fibers’ contents. The increase in PMF also resulted in the higher antioxidant capacity of biscuits. Such results indicate that pequi mesocarp is a promising raw material to be converted into functional flour to generate gluten‐free bakery products. Using pequi mesocarp to produce traditional and innovative food products is an important alternative to converting it into a value‐added raw material while decreasing waste and preventing environmental pollution. Also, this approach might contribute to the local economy by strengthening family farming and offering consumers functional food options, mainly for those who require gluten‐free diets.

One of the challenges in marketing PMF is its characteristic flavor and odor. When used in high concentrations (especially above 30%), it may not be well accepted by all consumers, particularly in markets where pequi is not a traditional ingredient. This occurs because some compounds in pequi flour may taste bitter if used in high concentrations. Some modifications are needed in the preparation of the cookies when higher quantities of flour are added, such as using flavorings or sweeteners to balance the flavor. Further studies are important to study the sensory aspects, including consumer preference, acceptance, and buying intention. Such evaluations will identify the acceptance of darker colors and higher firmness of consumers and confirm the potential of PMF application in biscuit production. Furthermore, it is essential to determine the shelf life/due date through the stability of the biscuits over storage, and the most suitable packaging.

## AUTHOR CONTRIBUTIONS

**Viviane Patrícia Romani**: Conceptualization; writing—original draft; methodology; writing—review and editing; project administration; supervision; data curation. **Daniel Emanuel Cabral de Oliveira**: Conceptualization; methodology; supervision; project administration; resources. **Juliana Aparecida Célia**: Methodology; formal analysis; data curation; investigation. **Adrielle Borges de Almeida**: Investigation; methodology; formal analysis; data curation. **Marcos Antônio Almeida Rocha**: Investigation; methodology; formal analysis. **Danihanne Borges e Silva**: Investigation; methodology; formal analysis. **Osvaldo Resende**: Methodology; project administration; supervision; resources.

## CONFLICT OF INTEREST STATEMENT

The authors declare no conflicts of interest.

## Supporting information



Supporting Information
