# Supplementary figures and images for: Exploring pequi (Caryocar brasiliense Camb.) mesocarp flour of Brazilian Cerrado biome to produce gluten‐free antioxidant biscuits
Source: J Food Sci. 2025 Mar 7;90(3):e70070. doi: 10.1111/1750-3841.70070 (PMC11924872; doi:10.1111/1750-3841.70070)

**Supporting information**

**Figure S1.** Scanning electron microscopy (SEM) of pequi mesocarp flour.
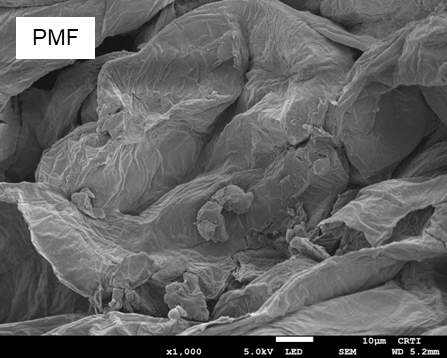

Supplement: Supplementary file 1 — Supporting Information [file JFDS-90-0-s001.docx]
